# Synthesis and Characterization of Novel Dendrons Bearing Amino-Nitro-Substituted Azobenzene Units and Oligo(ethylene glycol) Spacers: Thermal, Optical Properties, Langmuir Blodgett Films and Liquid-Crystalline Behaviour

**DOI:** 10.3390/molecules18021502

**Published:** 2013-01-25

**Authors:** Jesús Ortíz-Palacios, Efraín Rodríguez-Alba, Mayra Avelar, Ana Martínez, Maria del Pilar Carreón-Castro, Ernesto Rivera

**Affiliations:** 1 Instituto de Investigaciones en Materiales, Universidad Nacional Autónoma de México, Ciudad Universitaria, C.P. 04510 D.F., Mexico; 2 Instituto de Ciencias Nucleares, Universidad Nacional Autónoma de México, Ciudad Universitaria, C.P. 04510 D.F., Mexico

**Keywords:** dendron, azobenzene, optical properties, liquid crystals, Langmuir-Blodgett films

## Abstract

In this work, we report the synthesis and characterization of a novel series of first and second generation Fréchet type dendrons bearing amino-nitro substituted azobenzene units and tetra(ethylene glycol) spacers. These compounds were fully characterized by FTIR, ^1^H and ^13^C-NMR spectroscopies, and their molecular weights were determined by MALDI-TOF-MS. The thermal properties of the obtained dendrons were studied by TGA and DSC and their optical properties by absorption spectroscopy in solution and cast film. Molecular calculations were performed in order to determine the optimized geometries of these molecules in different environments. Besides, Langmuir and Langmuir Blodgett films were prepared with the first generation dendrons that were shown to be amphiphilic. Finally, some of the dendrons showed a liquid crystalline behaviour, which was studied by light polarized microscopy as a function of the temperature in order to determine the transition temperatures and the structure of the mesophase.

## 1. Introduction

Nowadays, dendrimers and dendrons are considered one of the most attractive research fields in polymer chemistry, due to their sophisticated structures and potential applications [1,2,3,4]. These molecules can be modified by introducing functional groups and specific units at different levels of their structure: core, branches or surface [5], giving rise to well-defined and highly functionalized molecules. Depending on the type of functional groups present in dendrimers, various properties have been already studied such as response to light, which has a wide variety of potential applications. Many reviews include the first examples of photo-responsive dendrimers [6,7,8,9] taking into account various examples of azo-dendrimers. The most recent review covering the most important aspects of azobenzene-containing dendrons and dendrimers reported until 2009 has been published by Caminade and Deloncle [10].

In the beginning azobenzenes had been exclusively incorporated as terminal groups of dendrimers and dendrons; the first examples were described by Vögtle and co-workers [11]. The first reported structures were obtained from poly(propyleneimine) (PPI) dendrimers built from either ethylenediamine [12] or 1,4-diaminobutane [1,2,3,4,5,6,7,8,9,10,11,12,13,14] cores. Except for the first example, all the terminal groups were generally azobenzenes [15,16,17,18].

The most popular types of dendrimers: poly(amidoamine) (PAMAM) dendrimers [19] and poly(arylether) dendrimers [20] have been rarely used as supports for azobenzene moieties. The first example of Fréchet-type azo-dendrimers was prepared by grafting through their core poly(arylether) dendrons bearing a single azobenzene group on the surface, leading to original dendrimers [21,22], having azobenzene units as terminal groups. Other types of poly(ether) dendrimers having long alkyl chains and azobenzene groups from generation 0 to generation 3 have been reported in the literature [23].

Unlike dendrimers, dendrons have been infrequently functionalized with azobenzene units on their surface. The first example was synthesized to be used as building block for dendrimers [21,22]. Also, more sophisticated systems such as polyether dendrons linked to a fullerene as core have been prepared [24].

Rau classified azobenzenes into three main groups, based on their photochemical behaviour [25]. Unsubstituted photochromic azobenzenes makes up the first group, known as “azobenzenes”. The thermally stable *trans* isomer exhibits a strong π-π* transition at 350 nm and a weak n-π* transition at 440 nm, whereas the *cis* isomer undergoes similar transitions but with a more intense n-π* band. In addition, “azobenzenes” have a relatively poor π-π* and n-π* overlap. The second group, known as “aminoazobenzenes” typically includes azobenzenes that are substituted by an electron-donor group and are characterized by the overlapping of the π-π* and n-π* bands. Finally, azobenzenes bearing both electron-donor and electron-acceptor groups belong to the third category, “pseudostilbenes”, where the π-π* and n-π* bands are practically superimposed and inverted on the energy scale with respect to the “azobenzenes” bands [25].

When donor-acceptor substituted azobenzenes are incorporated into a polymer backbone or side-chain, they provide very versatile materials from the applications point of view. In particular, “pseudostilbene” azobenzenes undergo rapid *trans-cis-trans* photoisomerization when they are irradiated with linear polarized light. The use of polarized light allows the selective activation of “pseudostilbenes” with polarization axis parallel to the absorbing radiation [26,27,28,29,30,31,32]. Azobenzene molecules are also known to undergo chromic changes through aggregation in various media including solution, spin-cast films and Langmuir-Blodgett layers. Both H-type and J-type aggregates have been observed [33]. On the other hand, azobenzene and poly(ethylene glycol) have been employed in the synthesis of amphiphilic azo-dyes, copolymers [34,35], nanomaterials [36,37], cellulose derivatives [38,39] and cyclodextrin polymers [40,41], sometimes forming supramolecular complexes with interesting properties [42]. Poly(ethylene glycol) segments provide flexibility and water solubility to the systems to which they are incorporated [43,44].

Previously, we have published the synthesis and characterization of four novel azo-dyes bearing terminal hydroxyl groups (**RED-PEG** series), the preparation of grafted azo-polymer films containing oligo(ethylene glycol) segments (**AC-g-PE-RED-PEG** series) [45], and the synthesis and characterization of a new series of azo-polymers bearing **RED-PEG** units in their structure (**pnPEGMAN** series) [46]. Very recently, we reported the synthesis and characterization of a series of liquid crystalline dyes bearing two amino-nitro-substituted azobenzene units linked by well defined oligo(ethylene glycol) spacers (**DIRED-PEG** series) [47].

In the last years, our research group has worked on the synthesis and characterization of amphiphilic azo-dyes and azo-polymers bearing oligo(ethylene glycol) segments with different architectures. Herein, we report the incorporation of the **RED-PEG** dyes into Fréchet type dendrons in order to obtain new liquid crystalline materials bearing azobenzene units. The thermal and optical properties of these dendrons were studied in detail. Some of them exhibited a liquid crystalline behaviour that was studied by DSC and Light Polarized Microscopy as a function of the temperature. Finally, the viability of these compounds to form Langmuir and Langmuir-Blodgett films was also investigated.

## 2. Results and Discussion

### 2.1. Synthesis of the Dendrons

**Scheme 1:**
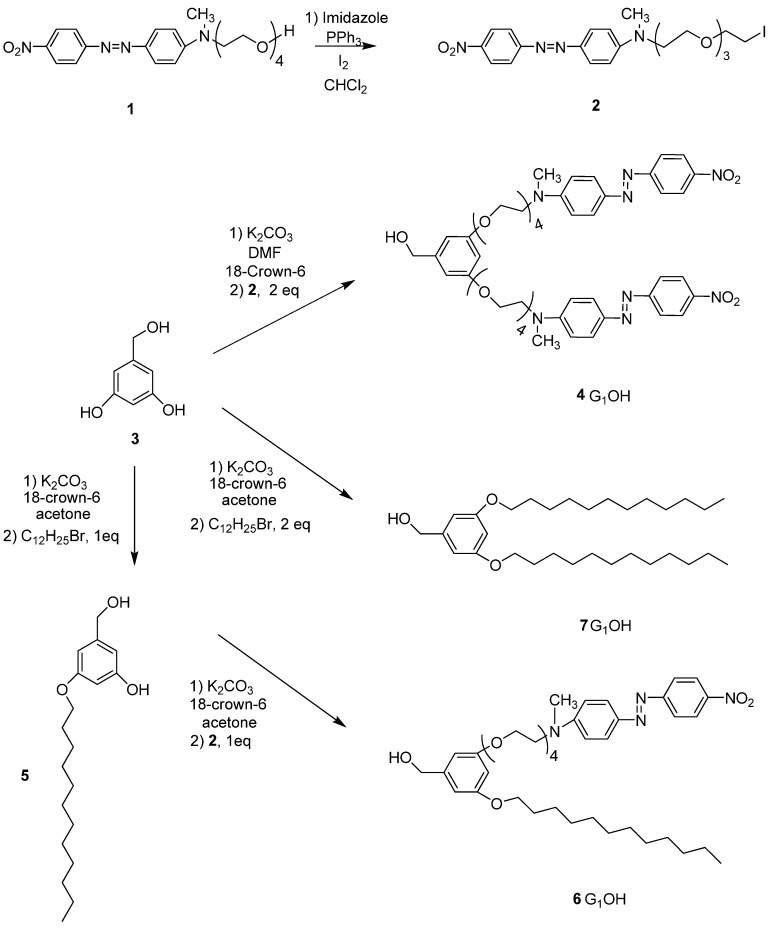
Synthesis of the first generation dendritic molecules.

**Scheme 2:**
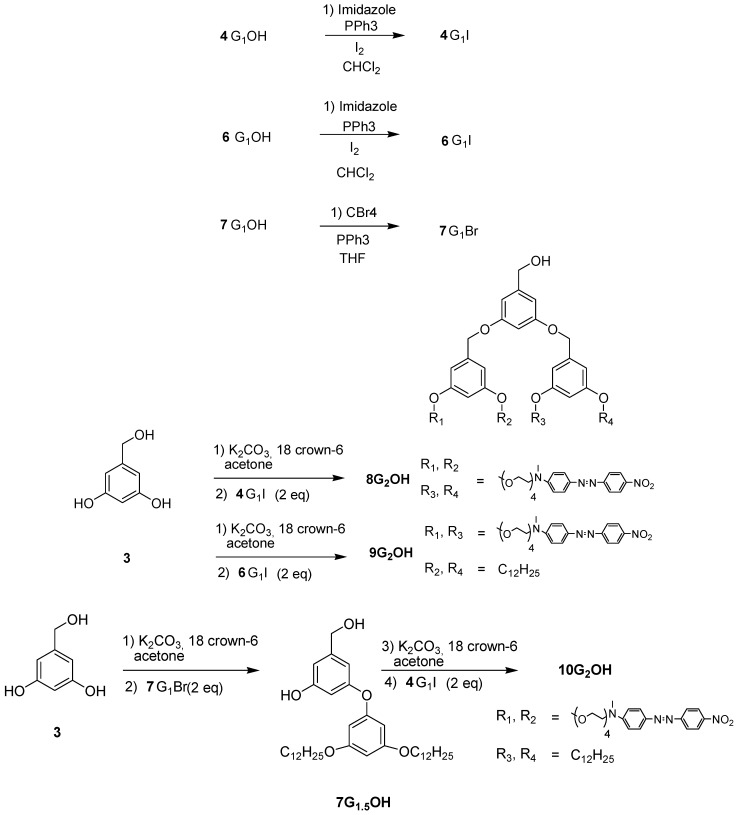
Synthesis of the second generation dendritic molecules.

Six novel Fréchet type dendrons bearing azobenzene groups have been synthesized using 3,5 dihydroxybenzylic alcohol as building unit, using the classical methodology described in the literature [45,48]. First and second generation dendritic molecules were prepared according to the synthetic sequences illustrated in Scheme 1, Scheme 2, respectively.

First **RED-PEG-4** (**1**) was treated in the presence of iodine and imidazole to give the corresponding alkyl iodide **2**. Then 3,5-dihydroxybenzylic alcohol (**3**) was reacted with **2** using K_2_CO_3_ as base and DMF as solvent, in the presence of 18-crown-6 to give the first generation dendron **4G_1_OH**. On the other hand, when **3** was treated with 1-dodecyl bromide (1 eq) under the same reaction conditions it gave the monoalkylated compound **5. **This intermediate was reacted with **2 **(1 equiv.) in the presence of K_2_CO_3_, DMF and 18-crown-6 to give **6G_1_OH**. Similarly, **3** was reacted with 2 equiv. of 1-dodecyl bromide under the same reaction conditions to yield the symmetric compound **7G_1_OH**.

First generation dendron **4G_1_OH** and **6G_1_OH** were treated with iodine and imidazole to give the corresponding halogenated compounds **4G_1_I** and **6G_1_I**. In the case of **7G_1_OH**, this compound was brominated in the presence of CBr_4_ and triphenylphosphine to give **7G_1_Br**. Once the activated first generation dendrons were obtained, **3** was reacted with 2 eq of **4G_1_I** or **6G_1_I **in the presence of K_2_CO_3_ and 18-crown-6 using DMF as solvent, to give the corresponding symmetric second generation dendritic molecules **8G_2_OH **and **9G_2_OH**, respectively. Similarly, **3** was reacted first with **7G_1_Br **and then with **4G_1_I **under the same reaction conditions to give the asymmetric dendron **10G_2_OH**.

### 2.2. Characterization of the Dendrons

All dendritic compounds were fully characterized by FTIR, ^1^H- and ^13^C-NMR spectroscopies and their molecular weights and purity were confirmed by MALDI-TOF-MS. The spectroscopic characterization of these compounds and that of the intermediates involved in the synthesis is included in the Experimental part, and in this section we explain in detail the assignment of the signals only for **4G_1_OH **and **8G_2_OH**.

The FTIR spectra of the azo-dendrons (not shown) were quite similar and exhibited the signals corresponding to the functional groups present in the molecules. For instance **4G_1_OH **and **8G_2_OH **exhibited a series of signals at 3442 (O-H), 2920, 2852 (C-H), 1594 (C=C), 1514, 1445 (N=N), 1375 (C-H), 1336 (NO_2_), 1292, 1102, 1067 (C-O of aryl and alkyl ethers), 857 and 826 (=C-H out of plane) cm^−1^.

**Figure 1:**
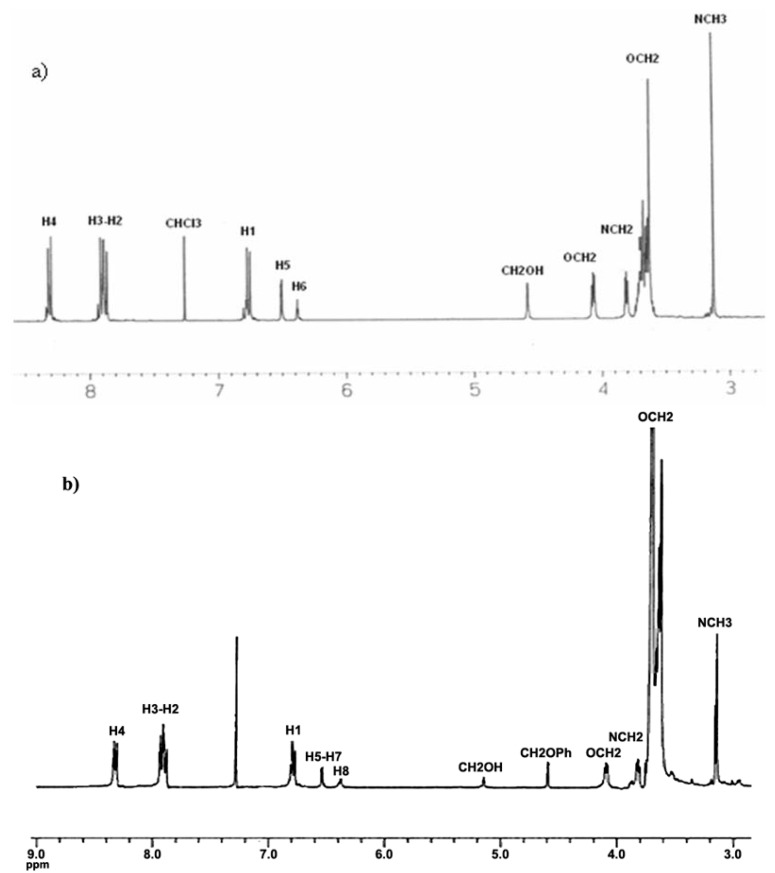
^1^H-NMR of: (**a**) First generation dendron **4G_1_OH** and (**b**) Second generation dendron **8G_2_OH**.

The ^1^H-NMR spectrum of **4G_1_OH **is shown in Figure 1a (the assignment of the signals is indicated in the experimental part). As we can see, in the aromatic region there are five signals corresponding to the protons present in the azobenzene unit and the phenyl group, which appear at 8.31 ppm (H^4^), 7.93 ppm (H^3^), 7.88 ppm (H^2^), 6.77 ppm (H^1^), 6.51 ppm (H^5^) and 6.37 ppm (H^6^). In the aliphatic region, we can observe five signals: a singlet at 4.59 ppm (CH_2_-OH), two triplets at 4.06 ppm and 3.82 ppm corresponding to protons OCH_2_ and NCH_2_), followed by a multiplet at 3.68 ppm due to the protons of all other OCH_2_ present in the tetra(ethylene glycol) segments. Finally, a singlet related to the methyl groups NCH_3_ was observed at 3.06 ppm.

In the ^13^C-NMR spectrum of this dendron (not shown) (the assignment of the signals is indicated in the experimental part), we can observe 12 signals in the aromatic region at 160.20, 157.50, 152.7, 147.50, 143.87, 143.54, 126.05, 124.56, 122.52, 111.61, 106.74, 101.10 ppm due to the 12 types of aromatic carbons present in the structure. In the aliphatic zone, we can see a series of signals at 71.07, 71.04, 70.64, 69.75, 68.57 ppm corresponding to all OCH_2 _carbons present in the tetra(ethylene glycol) segment. Finally, we can observe three more signals at 67.56 (CH_2_-OH), 52.16 (CH_2_N) and 39.10 ppm (CH_3_N). The molecular weight of **4G_1_OH** was confirmed by MALDI-TOF mass spectrometry and the base peak was observed at *m/z* = 969.5 as expected.

The ^1^H-NMR spectrum of **8G_2_OH** is shown in Figure 1b (the assignment of the signals is indicated in the experimental part). In the aromatic region, one can see six signals at 8.34 ppm (H^4^), 7.95 ppm (H^3^), 7.89 ppm (H^2^), 6.87 ppm (H^1^), 6.54 ppm (H^5^-H^7^), 6.41 ppm (H^8^), corresponding to the protons present in the azobenzene units and the phenyl groups of the dendron. In the aliphatic zone, we can observe two singlets at 5.14 ppm (CH_2_OH) and 4.59 ppm (CH_2_OPh), followed by two triplets at 4.08 and 3.81 ppm due to protons OCH_2_ and NCH_2_, respectively. Finally, a multiplet at 3.66 related to protons OCH_2_ present in the tetra(ethylene glycol) segment as well as a singlet at 3.14 ppm due to the NCH_3_ groups, were also seen.

The ^13^C-NMR spectrum of this dendron (not shown) (see experimental part) exhibits 16 signals in the aromatic zone at 160.15, 160.12, 156.89, 152.63, 147.48, 143.95, 143.46, 143.43, 126.05, 124.89, 122.79, 111.98, 105.98, 105.90, 101.11, 100.02 ppm, due to the 16 types of aromatic carbons present in the structure of the dendron. Moreover, in the aliphatic zone, we can observe different signals at 71.01, 70.99, 69.98, 68.82 and 67.87 ppm, due to carbons (CH_2_O) present in the tetra(ethylene glycol) segments. Finally, 3 more signals were perceived at 65.98 ppm (CH_2_OH), 52.18 ppm (CH_2_N) and 39.98 ppm (CH_3_N). The molecular weight of **8G_2_OH** was confirmed by MALDI-TOF mass spectrometry and the base peak was clearly observed at *m/z* = 2042.2, as expected.

### 2.3. Thermal Properties of the Dendritic Molecules

**Figure 2:**
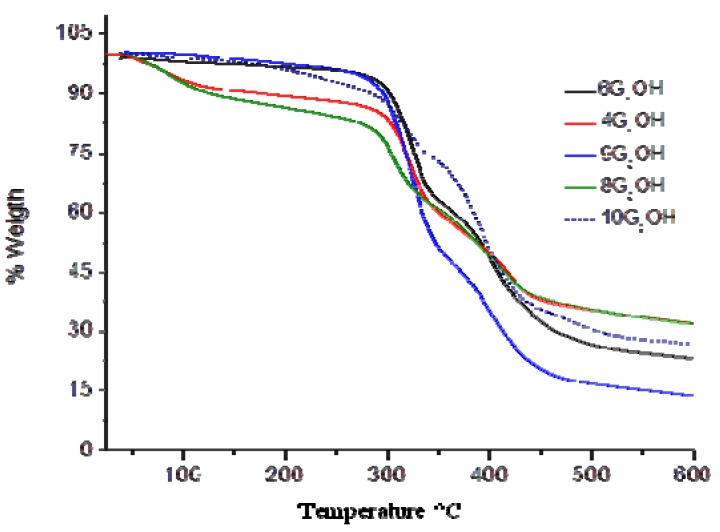
TGA of the obtained dendritic compounds.

The thermal properties of the obtained dendritic compounds were studied by Thermogravimetric Analysis (TGA) and Differential Scanning Calorimetry (DSC), and the results are summarized in Table 1. TGA curves of the dendrons are shown in Figure 2. All dendritic compounds exhibited good thermal stability, with T_10_ values ranging from 123 and 302 °C, and showed drastic degradation between 270 and 470 °C. As we can observe, dendrons **4G_1_OH** and **8G_2_OH **containing exclusively peripheral azobenzene groups are more susceptible towards degradation than those containing also side alkyl chains. We believe that this can be due to the presence of intramolecular H-aggregates resulting from the proximity of the azobenzene units, which make these compounds more succeptible towards degradation. After thermal fragmentation an azobenzene aggregate is more stabilized than a non-paired azobenzene unit. In contrast, the other dendritic compounds showed a higher thermal stability, which slightly decreases as the number of azobenzene units present in their structure augments. That is why first generation dendrons were shownto be thermally more stable than those of second generation. If we make a comparison between second generation dendrons **9G_2_OH** and **10G_2_OH**, it is worth pointing out that the latter, where the azobenzene groups are located in the same branch, showed a lower thermal stability. This fact revealed the formation of H-aggregates, which are more susceptible towards degradation than azobenzene units themselves. The presence of such kind of aggregates was further confirmed by absorption spectroscopy in solid state (*vide infra*).

**Table 1:** Thermal and optical properties of the dendrons.

Dendrons	T_10_ (°C) ^a^	Tm (°C) ^a^	λ_max_ (nm) ^b^	Cut off (nm) ^b^	Dipole Moment μ (D) [M062x]	Dipole Moment μ (D) [BPW91]
6G_1_OH	302	36	478	628	14.5253	13.9576
4G_1_OH	172	71	482	610	9.4828	9.1611
9G_2_OH	295	29	480	615	29.9523	29.4126
8G_2_OH	124	68	480	602	17.2420	15.5955
10G_2_OH	283	51	476	624	17.0627	15.7889

(a) Under nitrogen atmosphere using a heating rate of 10 °C/min; (b) In chloroform solution at room temperature.

**Figure 3:**
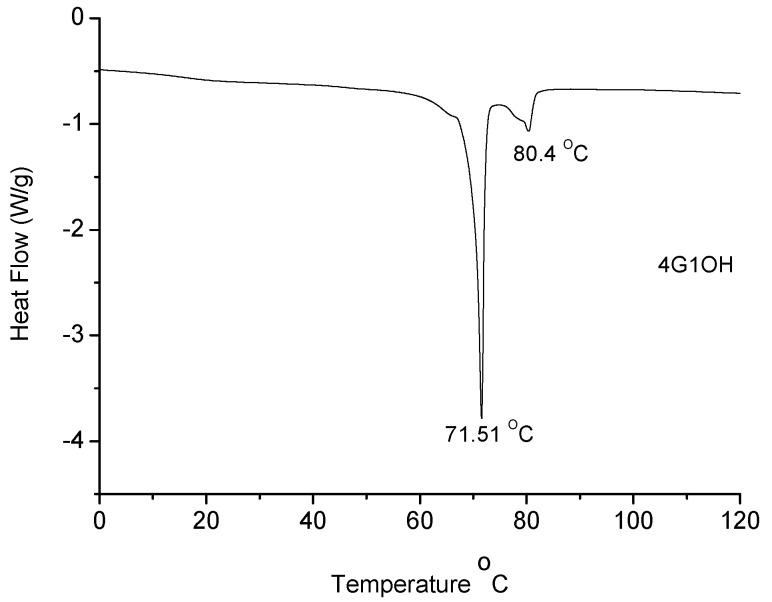
DSC of dendron **4G_1_OH**.

Melting points (T_m_) of the dendritic compounds were determined by DSC and the values are included in Table 1. All compounds exhibited T_m_ values between 29 and 71 °C. In particular **4G_1_OH** (Figure 3), exhibited a well defined mesophase, which clearly indicates the presence of liquid-crystalline domains.

DSC of **6G_1_OH**, **9G_2_OH**, **8G_2_OH** and **10G_2_OH** (not shown) exhibited broad asymmetric endotherms at 36, 30, 68 and 51 °C, respectively. The broadness of the curves indicates the presence of a discrete mesophase, which appears prior to the melting point. This behaviour was confirmed by repeating the DSC experiments after cooling down at a heating rate of 10 °C/min. A similar broadness in the DSC curves was reported in the literature for some dendrons containing azobenzene units, triazole, alkyl chains and poly(ethylene glycol) segments in their structure [63]. In the particular case of **4G_1_OH **(Figure 3), a well defined endotherm can be observed at 71 °C, revealing the presence of mesophase, followed by a second endotherm at 80 °C, due to the melting point of the dendron. In contrast, **9G_2_OH **only showed a symmetric endotherm at 30 °C, which is related to the melting point of this compound. In this particular case, no mesophase was detected because despite the presence of an azobenzene unit, the long oligo(ethylene glycol) spacers jointly with the alkyl chains make the structure of this dendritic compound too flexible to favour the formation of liquid crystalline domains. 

### 2.4. Liquid Crystalline Behaviour of the Dendrons

**Figure 4:**
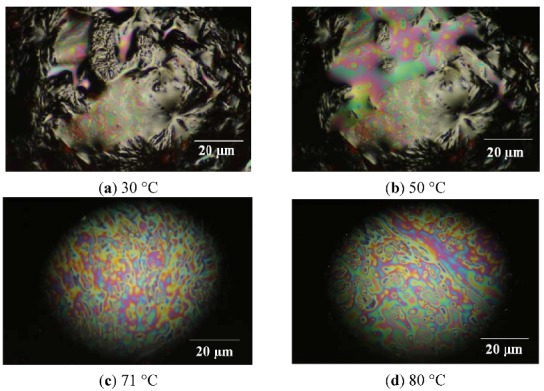
Liquid crystalline behaviour monitored by Light Polarized Microscopy Images for dendron **4G_1_OH**.

In order to confirm the liquid-crystalline (LC) behaviour of **4G_1_OH**, light polarized optical microscopy studies in function of the temperature were carried out [64,65] with a heating rate of 1 °C/min. Thus, the samples were gradually heated, which allowed us to observe LC behaviour in the range of temperatures of the mesophase. The images along the heating process are illustrated in Figure 4. 

Along gradual heating, the formation of structural arrangements was seen in the range of temperatures of the mesophase. For **4G_1_OH** (Figure 4 picture a, T = 30 °C), we can notice the presence of a crystalline structure. After heating, the dendron starts to soften and exhibits the presence of coloured domains (picture b, T = 50 °C). Furthermore, in the range of temperatures of the mesophase, we can notice the appearance of a LC phase with a well defined structure (picture c, T= 71°C), which persist until melting point was reached at 80 °C (picture d). Beyond this temperature, **4G_1_OH** passes from the LC to the isotropic phase, so that only darkness can be observed (not shown). During the cooling process this dendron adopts again a LC structure and recrystallises again at 40 °C. Experiments performed with light polarized microscopy allowed us to detect the C-LC and CL-I transitions for this compound at 71 °C and 82 °C, respectively. 

### 2.5. Optical Properties of the Dendrons

**Figure 5:**
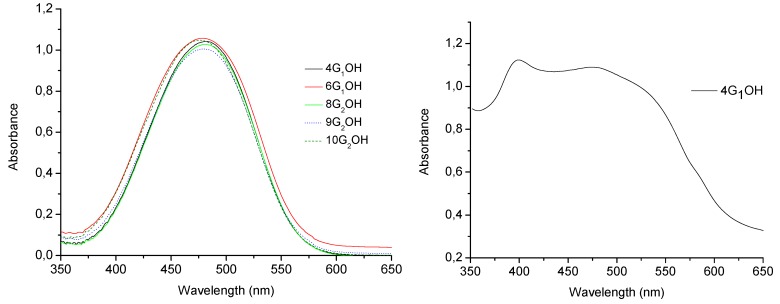
Absorption spectra of the obtained compounds: in solution (**left**) and in cast film (**right**).

Absorption spectra of the obtained compounds in chloroform solution are shown in Figure 5 and their optical properties are summarized in Table 1. All these compounds except **7G_1_OH** showed maxima absorption wavelength in the range between λ = 476–482 nm in CHCl_3_ solution [32,43,44,45,46,47]. Since these compounds contain high dipole moment azobenzene units in their structure, they exhibit the typical photochemical behaviour of azobenzenes belonging to the “pseudostilbenes” category. According to Rau, these compounds exhibit a total overlap of the π-π* and n-π* bands, which are inverted in the energy scale, so that only one band can be observed in their absorption spectra [25,32]. In fact, the higher the generation of the dendron is the more red-shifted the absorption band appears, which is an indication of the high conjugation degree in these molecules. On the other hand, in solid state these dyes behave differently. The absorption spectra of all these compounds in cast film are very similar. The UV-vis spectrum of **4G_1_OH** in cast film is shown in Figure 5b.

As we can see, the absorption spectra of the dendritic molecules in cast film showed a bathochromic shift of the maxima absorption bands. Besides, we can observe the presence of additional blue-shifted bands at ca. λ = 400 nm for both **4G_1_OH **and **6G_1_OH**, which reveals the presence of H-aggregates (Figure 5b, parallel interactions) [33]. A similar behaviour was observed with the second generation dendrons **9G_2_OH** and **10G_2_OH**. In this case, an additional blue shifted band appears at higher wavelength values due to formation of H-aggregates between the azobenzene chromophores [33,64]. Moreover, in the absorption spectra of these compounds in cast film, we can notice the presence of red-shifted absorption tails due to the presence of traces of J-aggregates (Figure 5b, head to tail interactions) [33].

### 2.6. Theoretical Results

**Figure 6:**
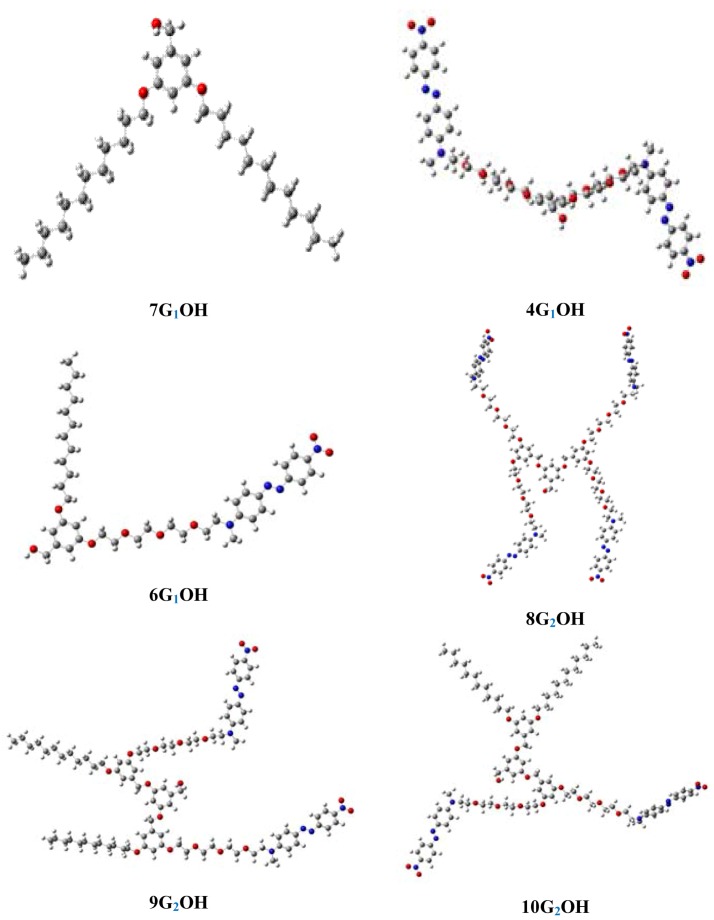
Optimized geometry of the first and second generation dendritic molecules in solution.

Optimized structures of the dendrons in chloroform solution are shown in Figure 6. As we can see in the case of dendrons bearing more than one azobenzene units, these chromophores stay away from each other, which avoid the formation of aggregates in solution. This results match well with those observed in the absorption spectra of the dendrons in chloroform solution. The dihedral angles (C-C-N-C) are equal to 90 degrees in **4G_1_OH**, **8G_1_OH**, **9G_2_OH** and **10G_2_OH** whereas **6G_1_OH** presents dihedral angles close to 180 degrees. For **7G_1_OH** all the carbon atoms of the aliphatic chains are quasi-linear due to the sp^3^ hybridization.

Theoretical UV-Vis spectra of the dendrons were calculated and the predicted λ_max_ values are listed in Table 2. Moreover, available experimental results are included for comparison. As it can be seen, there is a very good agreement between theoretical and experimental results. With these data it is possible to say that the methodology is appropriate and the structures correspond with the experimental ones. Analyzing the values, the most important difference is the change in the λ_max_ of the compounds with and without nitrogen functional groups. The molecule without azobenzene (**7G_1_OH**) is colorless and the λ_max_ appears in the UV region. In contrast, azobenzene compounds present a λ_max_ located in the blue region of the visible spectrum. It is important to note that there is no effect of the azobenzene content of the molecule since all the azobenzene units exhibit almost the same λ_max_ value.

**Table 2:** UV-vis λ_max_ in nm obtained at M062x/LANL2DZ level in chloroform are reported. Oscillator strengths (f) in parenthesis are included.

Dendrons	λ (f)	λ (exp)	Error (%)
7G_1_OH	189 (0.83)	-	-
4G_1_OH	469 (1.86)	482	3
6G_1_OH	470 (1.40)	478	3
8G_2_OH	469 (3.07)	482	3
9G_2_OH	469 (2.14)	480	2
10G_2_OH	471 (1.48)	476	1

Azobenzene dendrons have a small shoulder at lower λ close to the UV region with an absorption lambda similar to the value of the compound without nitrogen functional groups. Compound **6G_1_OH** present the most intense shoulder, while **10G_2_OH** present a weak intensity shoulder. Apparently, this second signal is correlated with the number of azobenzene units, *i.e.* and the intensity of the shoulder decreases as the number of azobenzene groups increases.

Table 3 reports the results of vertical ionization energy (I), vertical electron affinity (A) and the HOMO-LUMO gap (the energy difference between the highest occupied molecular orbital (HOMO) and the lowest unoccupied molecular orbital (LUMO)). The presence of the azobenzene groups in the dendrons generate a π-conjugated system that decreases slightly the ionization energy and increases dramatically the electron affinity. As a consequence, all azo-dendrons are better electron acceptors than compound **7G_1_OH**. The HOMO-LUMO gap is in agreement with the absorption spectra. Small values of the HOMO-LUMO gap indicate lower excitation energy and correspond to a greater λ values. 

**Table 3:** Ionization energy (I), electron affinity (A) and the HOMO-LUMO gap in eV.

Dendrons	7G_1_OH	4G_1_OH	6G_1_OH	8G_2_OH	9G_2_OH	10G_2_OH
I	6.8	6.2	6.1	6.1	6.1	6.1
A	0.2	3.4	3.4	3.4	3.4	3.4
HOMO-LUMO	7.9	4.1	4.1	4.1	4.1	4.1

The atomic charge of the azobenzene groups is analyzed and reported in Table 4. There is no difference in the atomic charge between the complexes. In all compounds, the N atoms have practically the same charge. The N atom of the tertiary amine is negative (N1), while one atom of the azo-group is negative (N3) and the other is positive (N2). Finally de N atom of the nitro group is positive (N4).

**Table 4:** Atomic charges.

	Atom	Atomic Charge
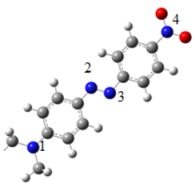	N1	−1.2
	N2	0.7
	N3	−0.7
	N4	1.6

### 2.7. Preparation of Langmuir and Langmuir-Blodgett Films

**Figure 7:**
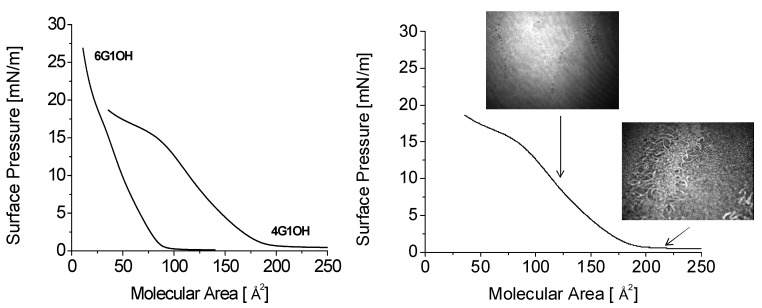
Isotherms of the Langmuir monolayers of **4G_1_OH **and **6G_1_OH** with BAM images.

It has been possible to prepare Langmuir films with some of these compounds and to plot the corresponding isotherms. Langmuir films of compounds **6G_1_OH **and **4G_1_OH** have been characterized by their surface pressure versus molecular area (π/A) isotherms and Brewster angle microscopy (BAM). The π/A isotherms obtained with **4G_1_OH** and **6G_1_OH **are shown in Figure 7a. The two main parameters of these isotherms, final molecular area *A_0_* extrapolated at zero pressure and collapse pressure π_c_, have been calculated from this graph. The good quality of the obtained films was confirmed by BAM images. In the film obtained with **4G_1_OH** (Figure 7b), when the molecular area reaches *A* ≈ 188 Å^2 ^(π_c _≈ 15 mN/m), a change of slope appears in the surface pressure curve, thereby indicating a greater compressibility of the film. At the same time, defects can be seen in the BAM picture showing that the film is collapsing at this point. In the case of **6G_1_OH** (not shown), the film is not continuous at large molecular areas and shows holes through which water can be seen. These domains smoothly weld together when the molecular area goes below *A* ≈ 100 Å^2^, and as long as the film does not enter the collapse regime only defectless surfaces are observed. This clearly indicates the formation of a homogeneous monomolecular layer.

**Figure 8:**
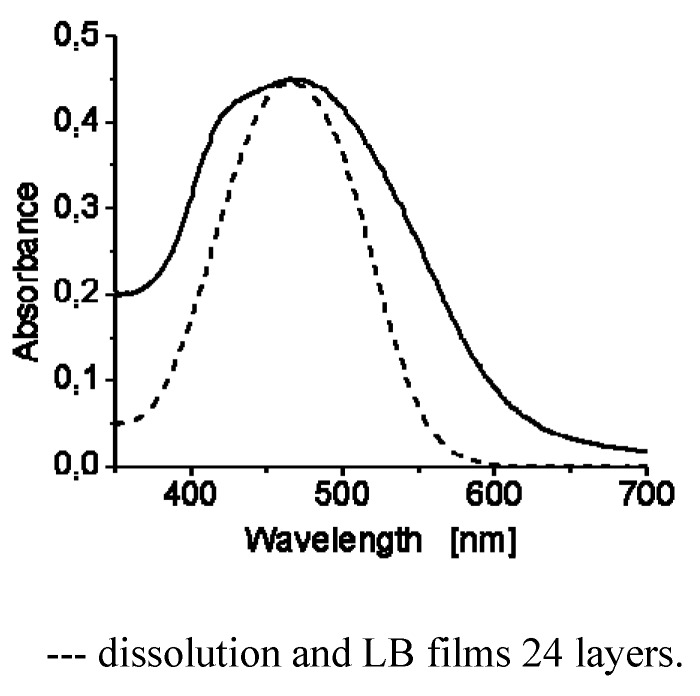
UV-Visible spectra of **4G1OH**.

Finally Langmuir-Blodgett films were prepared with **4G_1_OH** and **6G_1_OH** by transferring the monolayer on quartz slides. Film transfers were performed at surface pressures close to the collapse point, corresponding to the most condensed phase in the monolayer. The vertical dipping method with a dipping speed of 5 mm/min allowed us to get 2, 10, 14 and 24 multilayer films (Figure 8). The obtained multilayers films exhibited good quality and were analyzed by absorption spectroscopy. Figure 8 shows the UV-vis spectra of **4G_1_OH** in solution and LB film (24 layers). As we can observe the LB film of this dendron shows a maximum absorption wavelength at 468 nm. Moreover, we can perceive a blue shifted shoulder at λ = 425 nm, which reveals the formation of H-aggregates with the azobenzene units after the compression process. The amphiphilicity of **4G_1_OH** and **6G1OH** allows the preparation of LB films which can be useful for the future elaboration of NLO devices. 

## 3. Experimental

### 3.1. General Conditions

All reagents used in the synthesis of the dendrons were purchased from Aldrich and used as received without further purification. Acetone and dichloromethane were dried by distillation over calcium hydride. Precursor dye **RED-PEG-4** was synthesized according to the method previously reported by us [45] and the poly(aryl ether) dendrons were prepared as described in the literature [48]. 

FTIR spectra of the compounds were carried out on a Spectrum 100 (Perkin Elmer PRECISELY) spectrometer in solid state. ^1^H and ^13^C-NMR spectra of these compounds were recorded in CDCl_3_ solution at room temperature on a Bruker Avance 400 MHz spectrometer, operating at 400 MHz and 100 MHz for ^1^H and ^13^C, respectively.

Thermal properties of the obtained azo-dendrons were studied by determining T_10_ (10% weight loss temperature) and T_m_ (melting point). Thermogravimetric Analysis (TGA) was conducted on a Hi-Res TGA 2950 Instrument (from 20 to 800 °C) under inert atmosphere and Differential Scanning Calorimetry (DSC) was carried out in a DSC 2910 Instrument (from 20 to 250 °C), in both cases with a heating rate of 10 °C/min.

All dendritic compounds were dissolved in spectral quality solvents purchased from Aldrich, and their absorption spectra were recorded on a Varian Cary 1 Bio UV-vis (model 8452A) spectrophotometer at room temperature, using 1 cm quartz cuvettes. Absorption spectra of these compounds were also recorded in cast films, using the same instrument. Cast films were prepared from a saturated solution of the compounds in CHCl_3_, which was deposited over a glass substrate with further evaporation of the solvent.

### 3.2. Synthesis of the Dendrons

Precursor azo-dye **RED-PEG-4** (**1**) was prepared according to the method previously reported by us [45]. The synthesis of first and second generation dendritic molecules is shown in Scheme 1, Scheme 2, respectively. 

*3-Dodecyloxy-5-hydroxybenzyl alcohol* (**5**). A mixture of 1-bromododecane (1.12 g, 8.02 mmol), 3,5-dihydroxybenzyl alcohol (**3**, 1 g, 4.01 mmol), K_2_CO_3_ (4.43 g, 32 mmol) and 18-crown-6 in 500 mL of acetone was heated to reflux with vigorous stirring for 48 h. The reaction mixture was filtrated and concentrated at reduced pressure. The resulting product was purified by column chromatography using ethyl acetate/hexane 4:6, 5:5, and 6:4 as eluent to yield (**5**). Yield: 68%. ^1^H-NMR (CDCl_3_): δ (ppm) = 6.35 (s, 2H, H^1^-H^3^), 6.28 (s, 1H, H^2^), 4.46 (s, 2H, CH_2_OH), 3.80 (t, 2H, CH_2_OPh), 1.69 (m, 2H, CH_2_CH_2_OPh), 1.26 (m, 18H, other CH_2_ of the aliphatic chain), 0.88 (t, 3H, CH_3_) ppm. ^13^C-NMR (CDCl_3_): δ = 160.41 (1C, C^g^), 157.13 (1C, C^c^), 142.67 (1C, C^a^), 106.32 (1C, C^f^), 105.49 (1C, C^b^), 101.28 (1C, C^d^), 68.13 (1C, C^e^), 64.91 (1C, CH_2_OPh), 31.88 (1C, CH_2_CH_2_OPh), 29.61, 29.41, 29.32, 29.18, 25.98, 22.64 (9C, other CH_2_ of the aliphatic chain), 14.05 (1C, CH_3_) ppm. 


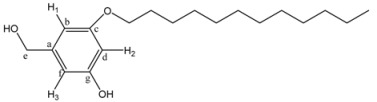


*(2-{2-[2-(2-Iodo-ethoxy)-ethoxy]-ethoxy}-ethyl)-methyl-[4-(4-nitro-phenylazo)-phenyl]-amine* (**2**). The intermediate **1 **was treated with imidazole (0.89 g, 13.1 mmol), triphenylphosphine (3.44 g, 13.1 mmol) and iodine (3.34 g, 13.1 mmol) in anhydrous dichloromethane (50 mL) at room temperature. The resulting solution was stirred for 6 h, filtered and concentrated at reduced pressure. The crude product was purified by column chromatography over silica gel using mixtures of ethyl acetate/hexane 4:6, 5:5 and 6:4 as eluent. Since this intermediate is very unstable it has to be immediately employed in the next reaction. Relative yield: 80% [the formation of this compound was confirmed by FTIR spectroscopy, due to the absence of the band at 3400 cm^−1 ^(OH) and the appearance of a new band at 600 cm^−1^(C-I)].

*[3-Dodecyloxy-5-(2-{2-[2-(2-{methyl-[4-(4-nitro-phenylazo)-phenyl]-amino}-ethoxy)-ethoxy]-ethoxy}-ethoxy)-phenyl]-methanol* (**6G_1_OH**). A mixture of **5** (0.706 g, 229 mmol), **2 **(1.14 g, 210 mmol), K_2_CO_3_ (1.58 g, 149 mmol) and 18-crown-6 dissolved in anhydrous DMF (50 mL) was heated at 80 °C with vigorous stirring for 48 h. The reaction mixture was filtered and evaporated at reduced pressure. The crude product was purified by column chromatography in silica gel using mixtures ethyl acetate/hexane 5:5 and 6:4 as eluent, to yield **6G_1_OH**. Yield: 62%. ^1^H-NMR (CDCl_3_): δ (ppm) = 8.24 (d, *J* = 9.02 Hz, 2H, H^4^), 7.84 (d, *J* = 9.05 Hz, 2H, H^3^), 7.81 (d, *J* = 9.21 Hz, 2H, H^2^), 6.70 (d, *J* = 9.23 Hz, 2H, H^1^), 6.44 (s, 2H, H^5^), 6.31 (s, 1H, H^6^), 4.53 (s, 2H, CH_2_OH), 4.03 (t, 2H, CH_2_-OPh), 3.84 (t, 2H, CH_2_N), 3.75 (t, 2H, OCH_2_R), 3.64-3.57 (m, 12H, all other OCH_2_), 3.06 (s, 3H, CH_3_N), 1.68 (m, 2H, OCH_2_CH_2_), 1.35 (m, 2H, OCH_2_CH_2_CH_2_), 1.30–1.19 (m, 16H, other CH_2_ groups of the dodecyl chain), 0.81 (t, 3H, CH_3_ of the aliphatic chain) ppm. ^13^C-NMR (CDCl_3_): δ = 160.63 (1C, C^k^), 160.22 (1C, C^m^), 156.89 (1C, C^e^), 152.67 (1C, C^a^), 147.53 (1C, C^h^), 143.92 (1C, C^d^), 143.37 (1C, C^i^), 126.08 (2C, C^c^), 124.62 (2C, C^g^), 122.59 (2C, C^f^), 111.60 (2C, C^b^), 105.60 (1C, C^j^), 105.26 (1C, C^n^), 101.04 (1C, C^l^), 70.88, 70.77, 69.80, 68.68, 68.22 (7C, all CH_2_O), 67.63 (1C, C^o^), 65.35 (1C, OCH_2_R), 52.31 (1C, CH_2_N), 39.26 (1C, CH_3_N), 31.91, 29.65, 29.62, 29.59, 29.39, 29.31, 26.06, 22.65 (10C, other CH_2_ of the aliphatic chain) 14.03 (1C, CH_3_ of the aliphatic chain) ppm. MALDITOF: C_40_H_58_N_4_O_8_ Calcd: 722.91 Found: (*m/z* = 722.47).


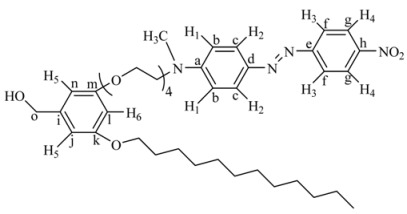


*[2-(2-{2-[2-(3-Dodecyloxy-5-iodomethyl-phenoxy)-ethoxy]-ethoxy}-ethoxy)-ethyl]-methyl-[4-(4-nitro-phenylazo)-phenyl]-amine* (**6G_1_I**)*.* **6G_1_OH** (0.22 g, 0.44 mmol) was treated with imidazole (0.055 g, 0.80 mmol), triphenylphosphine (0.21 g, 0.80 mmol) and iodine (0.20 g, 0.80 mmol) in anhydrous dichloromethane (50 mL) at room temperature. The resulting solution was stirred for 6 h, filtered and concentrated at reduced pressure. The crude product was purified by column chromatography in silica gel using a mixture of ethyl acetate/hexane (5:5 and 6:4) as eluent to give **6G_1_I**. Because of its instability this intermediate was immediately used in the next reaction. Relative yield: 65% (the formation of this compound was confirmed by FTIR spectroscopy). 

*3,5-Bis-[3-dodecyloxy-5-(2-{2-[2-(2-{methyl-[4-(4-nitro-phenylazo)-phenyl]-amino}-ethoxy)-ethoxy]-ethoxy}-ethoxy)-benzyloxy]-phenyl}-methanol* (**9G_2_OH**). 3,5-Dihydroxybenzyl alcohol (**3**, 0.043 g, 0.31 mmol) was reacted with **6G_1_I** (0.13 g, 0.015 mmol), K_2_CO_3_ (0.18 g, 1.2 mmol) and a catalytic amount of 18-crown-6 in dry acetone (50 mL). The reaction mixture was heated to reflux for 48 h, cooled to room temperature, filtered and concentrated at reduced pressure. The crude product was purified by column chromatography in silica gel using mixtures of ethyl acetate/hexane 8:2, 9:1 and pure ethyl acetate as eluent, to give **9G_2_OH**. Yield: 45%. ^1^H-NMR (CDCl_3_): δ = 8.28 (d, *J* = 9.10 Hz, 4H, H^4^), 7.89 (d, *J* = 9.01 Hz, 4H, H^3^), 7.87 (d, *J* = 9.30 Hz, 4H, H^2^), 6.75 (d, *J* = 9.30 Hz, 4H, H^1^), 6.57 (s, 2H, H^9^), 6.54 (s, 4H, H^7^-H^8^), 6.49 (s, 1H, H^6^), 6.40 (s, 2H, H^5^), 4.92 (s, 4H, PhOCH_2_Ph), 4.59 (s, 2H, CH_2_OH), 4.08 (t, 4H, OCH_2_), 3.90 (t, 4H, OCH_2_R), 3.81 (t, 4H, OCH_2_), 3.70–3.60 (m, 24H, other OCH_2 _and CH_2_N), 3.11 (s, 6H, CH_3_N), 1.74 (m, 4H, OCH_2_CH_2_R), 1.41 (m, 4H, OCH_2_CH_2_CH_2_R), 1.33–1.25 (m, 32H, other CH_2_ of the aliphatic chains), 0.87 (t, 6H, CH_3_ of the aliphatic chains) ppm. ^13^C-NMR (CDCl_3_): δ = 160.51 (2C, C^k^), 160.08 (2C, C^o^), 156.09 (2C, C^q^), 152.98 (2C, C^e^), 147.29 (2C, C^a^), 143.74 (2C, C^h^), 143.41 (2C, C^d^), 130.12 (2C, C^m^), 126.50 (4C, C^g^), 124.63 (4C, C^f^), 122.34 (4C, C^c^), 111.85 (4C, C^b^) , 106.03 (4C, C^n^-C^r^), 105.71 (2C, C^j^), 101.31 (2C, C^p^), 100.98 (1C, C^l^), 70.82 (2C, C^s^), 70.73, 70.69, 69.99 (6C, OCH_2_), 68.57 (1C, C^t^), 68.14, 67.63, 67.49, 65.51 (10C, all other OCH_2_), 52.37 (2C, CH_2_N), 39.43 (2C, CH_3_N), 31.88, 30.57, 29.63, 29.59, 29.58, 29.38, 29.30, 29.25, 26.03, 22.64 (22C, CH_2_ of the aliphatic chain), 14.06 (2C, CH_3_ of the aliphatic chain) ppm. MALDITOF: C_87_H_120_N_8_O_17_ Calcd: 1549.93 Found: (*m/z =* 1549.91). 


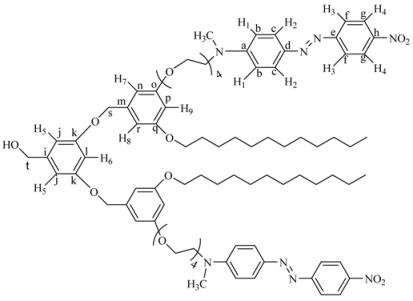


*(3,5-Bis-dodecyloxy-phenyl)-methanol* (**7G_1_OH)**. A mixture of 1-bromododecane (1 g, 4.01 mmol), 3,5-dihydroxybenzyl alcohol (**3**, 0.28 g, 2.0 mmol), K_2_CO_3_ (1.10 g, 8.0 mmol) and 18-crown-6 dissolved in 50 mL of acetone was heated to reflux with vigorous stirring for 48 h. The reaction mixture was filtered and concentrated at reduced pressure. The resulting product was purified by flash column chromatography in silica gel using mixtures ethyl acetate/hexane (1:9 and 2:8) as eluent to give **7G_1_OH**. Yield: 75%. ^1^H-NMR (CDCl_3_): δ = 6.49 (s, 2H, H^1^), 6.37 (s, 1H, H^2^), 4.61 (s, 2H, CH_2_OH), 3.93 (t, 4H, OCH_2_), 1.76 (m, 4H, OCH_2_CH_2_), 1.26 (s, 36H, other CH_2_ of the aliphatic chains), 0.88 (t, 6H, CH_3_) ppm. ^13^C-NMR (CDCl_3_): δ = 160.52 (2C, C^c^), 143.16 (1C, C^a^), 105.02 (2C, C^b^), 100.52 (1C, C^d^), 68.03 (1C, C^e^), 65.44 (2C, OCH_2_), 63.08 (2C, OCH_2_CH_2_), 32.79, 31.90, 29.58, 29.36, 29.25, 26.02, 25.7, 22.87, (18C, all other CH_2_), 14.09 (2C, CH_3_) ppm. 


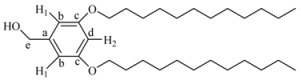


*1,3-Bis-dodecyloxy-5-iodomethyl-benzene* (**7G_1_Br**). **7G_1_OH** was treated with carbon tetrabromide (1.33 g, 4 mmol) and triphenylphosphine (1.07 g, 4 mmol) in dry THF (50 mL) at 0 °C and the resulting solution was stirred for 6 h at room temperature. The crude product was purified by column chromatography in silica gel using mixtures acetate/hexane 1:9 and 2:8 as eluent to give **7G_1_Br**. Because of its instability, this intermediate was used in the next step. Relative yield: 60% (the formation of this compound was confirmed by FTIR spectroscopy).

*3-(3,5-Bis-dodecyloxy-benzyloxy)-5-hydroxymethyl-phenol* (**7G_1.5_OH)***.* **7G_1_Br **(0.235 g, 0.43 mmol), 3,5-dihydroxybenzyl alcohol (**3**, 0.122 g, 0.87 mmol), K_2_CO_3_ (0.602 g, 4.3 mmol) and 18-crown-6 dissolved in 500 mL of acetone were heated to reflux with vigorous stirring for 48 h. The reaction mixture was filtered and concentrated at reduced pressure. The resulting product was purified by flash column chromatography in silica gel using mixtures ethyl acetate/hexane 3:7 and 4:6 as eluent to give **7G_1.5_OH**. Yield: 60%. ^1^H-NMR (CDCl_3_): δ = 6.39 (s, 1H, H^2^), 6.28 (s, 2H, H^3^), 6.26 (s, 2H, H^1^), 6.22 (s, 1H, H^4^), 5.17 (s, 2H, OCH_2_Ph), 4.70 (s, 2H, CH_2_OH), 4.35 (s, 4H, PhOCH_2_R), 3.77 (t, 4H, PhOCH_2_CH_2_R), 1.16 (m, 4H, CH_2_ of the aliphatic chain), 1.28 (m, 4H, CH_2_ of the aliphatic chain), 1.14 (m, 28H, other CH_2_ of the aliphatic chain), 0.76 (t, 6H, CH_3_) ppm. ^13^C-NMR (CDCl_3_): δ = 160.43 (1C, C^h^), 160.11 (1C, C^i^), 157.30 (2C,C^c^), 143.03 (1C, C^e^), 139.04 (1C, C^a^), 106.76 (1C, C^f^), 105.81 (1C, C^g^), 105.66 (2C, C^b^), 101.65 (1C, C^j^), 100.79 (1C, C^d^), 70.01 (1C, C^k^), 68.16 (2C, OCH_2_R), 64.96 (1C, C^l^), 53.43, 31.96, 29.72, 29.68, 29.64, 29.47, 29.39, 29.30, 26.09, 22.72 (20C, other CH_2_ of the aliphatic chains), 14.14 (2C, CH_3_) ppm.


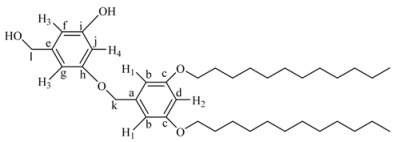


*[3,5-Bis-(2-{2-[2-(2-{methyl-[4-(4-nitro-phenylazo)-phenyl]-amino}-ethoxy)-ethoxy]-ethoxy}-ethoxy)-phenyl]-methanol* (**4G_1_OH**). A mixture of **2 **(1.3 g, 2.3 mmol), 3,5-dihydroxybenzyl alcohol (**3**, 0.11 g, 0.79 mmol), K_2_CO_3_ (0.44 g, 3.1 mmol) and 18-crown-6 dissolved in 50 mL of anhydrous DMF was heated at 80 °C with vigorous stirring for 48 h. Then, the reaction mixture was filtered and concentrated under vacuum. The crude product was purified by flash column chromatography in silica gel eluting first with a mixture ethyl acetate/hexane 9:1, and then with ethyl acetate 100% to give the first generation dendron **4G_1_OH**. Yield: 45%. ^1^H-NMR (CDCl_3_): δ = 8.31 (d, 4H, *J* = 9.06 Hz, H^4^), 7.93 (d, 4H, *J* = 9.06 Hz, H^3^), 7.88 (d, 4H, *J* = 9.32 Hz, H^2^), 6.77 (d, 4H, *J* = 9.32 Hz, H^1^), 6.51 (s, 2H, H^5^), 6.37 (s, 1H, H^6^), 4.59 (s, 2H, CH_2_OH), 4.06 (t, 4H, OCH_2_), 3.82 (t, 4H, NCH_2_), 3.68 (m, 24H, all OCH_2_), 3.06 (s, 6H, CH_3_N) ppm. ^13^C RMN (CDCl_3_): δ = 160.27(2C, C^k^), 157.44 (2C, C^e^), 152.70 (2C, C^a^), 147.55 (2C, C^h^), 143.91 (2C, C^d^), 143.65 (1C, C^i^), 126.05 (4C, C^c^), 124.56 (4C, C^g^), 122.52 (4C, C^f^), 111.61 (4C, C^b^), 106.74 (2C, C^j^), 101.10 (1C, C^l^), 70.90, 70.77, 70.53, 69.74, 68.57 (14C, all OCH_2_), 67.56 (1C, C^m^), 52.19 (2C, CH_2_N), 39.13 (2C, CH_3_N) ppm. MALDITOF: C_49_H_60_N_8_O_13_ Calcd: 969.05 Found: (*m/z =* 969.5). 


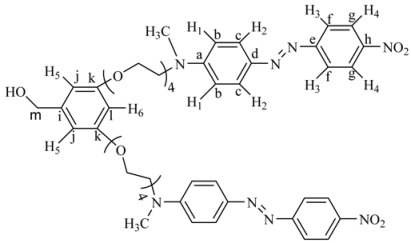


*(E)-N,N'-(2,2'-(2,2'-(2,2'-(2,2'-(5-(iodomethyl)-1,3-phenylene)bis(oxy) bis(ethane-2,1-diyl))bis(oxy)bis(ethane-2,1-diyl))bis(oxy)bis(ethane-2,1-diyl))bis(oxy) bis(ethane-2,1-diyl))bis(N-methyl-4-((E)-(4-nitrophenyl)diazenyl)aniline)* (**4G_1_I**). **4G_1_OH** (0.22 g, 0.44 mmol), imidazole (0.055 g, 0.80 mmol), triphenylphosphine (0.21 g, 0.80 mmol) and iodine (0.20 g, 0.80 mmol) were dissolved in 50 mL of anhydrous dichloromethane at room temperature. The resulting solution was stirred for 6 h, filtered and concentrated at reduced pressure. The crude product was purified by column chromatography in silica gel using a mixture of ethyl acetate/hexane 8:2 as eluent to give **4G_1_I**. Because of its instability this intermediate was immediately used in the next reaction. Relative yield: 60% (the formation of this compound was confirmed by FTIR spectroscopy). 

*{3-(3,5-Bis-dodecyloxy-benzyloxy)-5-[3,5-bis-(2-{2-[2-(2-{methyl-[4-(4-nitro-phenylazo)-phenyl]-amino}-ethoxy)-ethoxy]-ethoxy}-ethoxy)-benzyloxy]-phenyl}-methanol (***10G_2_OH**)*.* **7G_1.5_OH** (0.123 g, 0.02 mmol), **4G_1_I **(0.224 g, 0.022 mmol), K_2_CO_3_ (0.11 g, 0.082 mmol) and 18-crown-6 were dissolved in 50 mL of acetone and heated to reflux for 48 h. The reaction mixture was filtered and concentrated at reduced pressure. The crude product was purified by column chromatography in silica gel first eluting with a mixture ethyl acetate/hexane 8:2 and then with ethyl acetate 100%, to yield **10G_2_OH**. Yield: 70%. ^1^H-NMR (CDCl_3_): δ = 8.17 (d, *J* = 8.94 Hz, 4H, H^4^), 7.77 (d, *J* = 7.02 Hz, 4H, H^3^), 7.75 (d, *J* = 7.08 Hz, 4H, H^2^), 6.63 (d, *J* = 9.2 Hz, 4H, H^1^), 6.44 (s, 4H, H^8^-H^10^), 6.41 (s, 2H, H^5^-H^6^), 6.37 (s, 1H, H^7^), 6.27 (s, 2H, H^9^-H^11^), 4.80 (s, 4H, PhOCH_2_Ph), 4.47 (s, 2H, CH_2_OH), 3.95 (t, 4H, OCH_2_), 3.79 (t, 4H, OCH_2_R), 3.68 (t, 4H, NCH_2_), 3.57–3.49 (m, 24H, OCH_2_), 2.99 (s, 6H, CH_3_N), 1.63 (m, 4H, OCH_2_CH_2_R), 1.30 (m, 4H, CH_2_), 1.13 (m, 32H, other CH_2_ of the aliphatic chains), 0.75 (t, 6H, CH_3_) ppm. ^13^C-NMR (CDCl_3_): δ = 160.52 (2C, C^k^-C^m^), 160.08 (2C, C^w^), 160.01 (2C, C^q^), 152.65 (2c, C^e^), 147.34 (1C, C^a^), 143.68 (2C, C^h^), 143.54 (1C, C^s^), 139.27 (1C, C^o^), 138.94 (2C, C^d^), 126.22 (4C, C^g^), 124.66 (4C, C^c^), 122.54 (4C, C^f^), 111.59 (4C, C^b^), 106.00 (1C, C^j^), 105.76 (1C, C^n^), 105.67 (2C, C^t^), 101.33 (2C, C^p^), 101.12 (1C, C^u^), 100.76 (1C, C^r^), 100.00 (1C, C^l^), 70.85 (1C, C^v^), 70.74 (1C, C^x^), 70.70, 70.12, 69.89, 69.67 (8C, OCH_2_), 68.59(1C, C^y^), 68.10, 67.50, 65.21 (8C, all other OCH_2_), 52.25 (2C, CH_2_N), 39.38 (2C, CH_3_N), 31.93, 29.68, 29.65, 29.42, 29.36, 29.29, 26.08, 22.70 (20C, all other CH_2_ of the aliphatic chains), 14.13 (2C, CH_3_ of the aliphatic chains) ppm. MALDITOF: C_87_H_120_N_8_O_17_ Calcd: 1549.93 Found: (*m/z =* 1549.91).


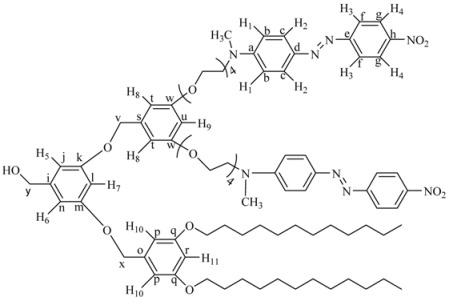


*(3,5-bis(3,5-bis(2-(4-((E)-(4-nitropheyl)diazenyl)phenyl)-5,8,11,14,17-pentaoxa-2-azanonadecan-19-yloxy)benzyloxy)phenyl)methanol* (**8G_2_OH**)*.* **3** (0.0704 g, 0.503 mmol) was reacted in the presence of **4G_1_I** **(**1.091 g, 1.106 mmol), K_2_CO_3_ (0.556 g, 4.02 mmol), DMF (10 mL) and 18-crown-6 as catalyst. The reaction was heated at 100 °C with vigorous stirring for 4 days. The crude product was dried under vacuum and purified by column chromatography in silica gel using chloroform 100% as eluent, increasing gradually the polarity until chloroform: acetone 97:3 to give **8G_2_OH** as a red powder. Yield: 15%. ^1^H-NMR (CDCl_3_): δ = 8.34 (d, 8H, *J* = 8.81 Hz, H^4^), 7.95 (d, 8H, *J* = 8.81 Hz, H^3^), 7.89 (d, 8H, *J* = 9.32 Hz, H^2^), 6.87 (d, 8H, *J* = 9.32 Hz, H^1^), 6.54 (s, 6H, H^5^-H^7^), 6.41 (s, 3H, H^6^-H^8^), 5.14 (s, 2H, CH_2_OH), 4.59 (s, 4H, PhCH_2_OPh), 4.18 (t, 8H, CH_2_O), 3.81 (t, 8H, CH_2_N), 3.64 (m, 48H, OCH_2_), 3.16 (s, 12H, CH_3_N) ppm. ^13^C-NMR (CDCl_3_): δ = 160.15 (4C, C^o^), 160.12 (2C, C^k^), 156.89 (4C, C^e^), 152.63 (4C, C^a^), 147.48 (4C, C^h^), 143.95 (4C, C^d^), 143.46 (1C, C^i^), 143.43 (2C, C^m^), 126.05 (8C, C^c^), 124.89 (8C, C^g^), 122.79 (8C, C^f^), 111.98 (8C, C^b^), 105.98 (2C, C^j^), 105.90 (4C, C^n^), 101.11 (1C, C^l^), 100.02 (2C, C^p^), 71.01 (2C, C^q^), 70.99 (1C, C^r^), 69.98, 68.82, 67.87, 65.98 (28C, all OCH_2_), 52.18 (4C, CH_2_N), 39.98 (4C, CH_3_N) ppm. MALDITOF: Calcd (C_105_H_124_N_16_O_27_): 2042.20 Found (*m/z =* 2042.20). 


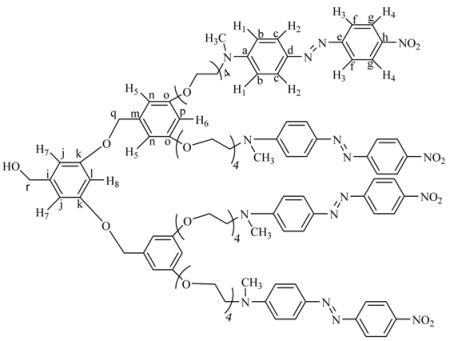


### 3.3. Computational Details

Density functional theory [49,50,51] as implemented in Gaussian09 [52] was used for all calculations. Full geometry optimizations of the obtained compounds without symmetry constraints and frequency analysis were carried out for all the stationary points, using the BPW91 density functional [53,54,55], and the LANL2DZ basis set [56,57,58]. Local minima were identified by the number of imaginary frequencies (NIMAG = 0). The absorption spectra of the dendritic molecules have been computed with time-dependent density functional theory (TD-DFT) using M062X functional [59] and the same basis sets. Theoretically, the intensity of the bands was expressed in terms of the oscillator strengths (f). Stationary points were first modeled in gas phase (vacuum), and solvent effects were included a posteriori, applying single point calculations at the same level of theory, using a polarizable continuum model, specifically the integral-equation-formalism (IEF-PCM) [60,61,62] with chloroform as solvent, in order to make a comparison with available experimental results. Vertical Ionization Energy (I) was calculated as the difference between the energy of the cation and the neutral molecule, assuming that both of these have the ground-state nuclear configuration of the neutral molecule. Vertical Electron Affinity (A) represents the energy difference between the neutral molecule and the anion, calculated with the ground-state nuclear configuration of the former.

### 3.4. Preparation of Langmuir and Langmuir-Blodgett Films

Solutions of the dendritic molecules were prepared by dissolving these compounds in chloroform, using concentrations of 1 mg/mL. These solutions were spread on the water surface with a microsyringe, and the film was left to equilibrate for 15–20 min before the compression started. Data were collected with a KSV 5000 system 3 using a Teflon trough and barriers in a dust-free environment, the isotherms were recorded at 20 °C and temperature was controlled to ± 0.1 °C. Ultrapure water (ρ = 18.2 MΩ•cm) obtained from a Milli-DIPAK/ Milli-Q185 ultrapurification system from Millipore was used as subphase. The monolayers were compressed at 5 mm/min. Langmuir-Blodgett films were obtained by transfer on quartz slides. The vertical dipping method with a dipping speed of 5 mm/min was used to obtain 2, 10, 14 and 24 multilayer films. Film transfers were preformed at surface pressures close to the collapse point, corresponding to the most condensed phase in the monolayer. The phase transitions of the spread monolayer were monitored using a Nanofilm Technologie Brewster Angle Microscope (BAM) fitted with a Teflon trough. Along the compression process, some images on the air-solution interface were taken with a CCD camera. The image contrast consisted of 256 gray levels.

## 4. Conclusions

Two novel series of first and second generation Frechet type dendritic compounds bearing amino-nitro-substituted azobenzene units and tetra(ethylene glycol) spacers were synthesized and characterized. These materials exhibited a good thermal stability with T_10_ values between 124–302 °C Showing drastical degradation between 270 and 470 °C All compounds bearing pseudostilbene type azobenzene units in CHCl_3_ solution exhibited maxima absorption wavelengths in the range of λ = 476–482 nm and no aggregation was observed, which is in agreement with the results and the optimized geometries obtained by molecular modelling. However, in cast film these molecules showed an additional absorption band at λ = 400 nm that indicates the formation of H-aggregates. Differential Scanning Calorimetry jointly with Light Polarized Microscopy studies revealed that dendron **4G_1_OH** exhibited a liquid crystalline behaviour, with the formation of a mesophase. Besides, **4G_1_OH** and **6G_1_OH** are able to form Langmuir films and Langmuir Blodgett multilayers due to their amphiphilic character and good compressibility.
